# Analysis of respiratory pathogens in pediatric acute respiratory infections in Lanzhou, Northwest China, 2019-2024

**DOI:** 10.3389/fcimb.2024.1494166

**Published:** 2025-01-17

**Authors:** Qian Wang, Yunyan Pan, Hongwei Gao, Youli Zhao, Xinzhe Gao, Yichen Da, Shaomin Niu, Chongge You

**Affiliations:** Laboratory Medicine Center, Lanzhou University Second Hospital, Lanzhou, China

**Keywords:** ARTIS, respiratory pathogens, prevalence, pediatric, NPIs

## Abstract

**Objective:**

This study aimed to assess the prevalence and distribution of respiratory pathogens in children under 18 years old with Acute Respiratory Infections (ARTIs) in Lanzhou, Northwest China, from July 2019 to January 2024.

**Methods:**

The respiratory pathogens studied were FluA, FluB, PIV, RSV, ADV, MP, CP, CB, and LP, detected by indirect immunofluorescence assay (IIF). Data were obtained from the laboratory information system (LIS) of the Lanzhou University Second Hospital. As in Lanzhou, NPIs were implemented in January 2020, and were lifted in December 2022, data were divided into pre-NPIs (July 2019 to December 2019), NPIs (January 2020 to December 2022) and post-NPIs (January 2023 to January 2024) periods for analysis. Pearson’s chi-square test, ANOVA, and Fisher’s exact test were used to evaluate statistical significance in variable differences, with P < 0.05 considered significant.

**Results:**

A total of 29,659 children diagnosed with ARTIs were included in the study, with 13030(43.93%) test positive for at least one pathogen. Single-pathogen infections predominated (33.10%), while co-detection of MP and PIV was the most common among multi-pathogen cases (52.96%). Pathogen detection rates were notably higher in female children (50.62%) and preschool-aged children (53.45%) and exhibited seasonal variations, with a pronounced increase in winter (47.61%) and a peak in November (48.92%). MP had the highest detection rate (38.59%), followed by PIV (10.18%). Detection rates significantly increased following the lifting of NPIs, rising from 33.82% (SD ± 13.13) during NPIs to 64.42% (SD ± 4.67) (P < 0.001), with 2023 showing the highest detection rate (64.61%) and largest participant count (9,591). In November 2023, detection rates reached their highest level at 73.09%. Post-NPI, most pathogens, except CB and LP, demonstrated significantly higher prevalence (P<0.001).

**Conclusion:**

In the Lanzhou region, MP and PIV were identified as the most prevalent respiratory pathogens among children with ARTIs, with peak detection rates during the winter season. Boys and school-age children exhibited higher susceptibility to these infections. NPIs played a critical role in reducing respiratory pathogen transmission. Once NPIs were lifted, a marked resurgence in pathogen incidence highlighted their impact on controlling infection spread.

## Introduction

Acute respiratory tract infections (ARTIs) represent a major public health challenge, resulting in high morbidity and mortality, especially among children, the elderly, and immunocompromised populations (Gottlieb, 2019). Each year, ARTIs accounted for approximately 1.3 million deaths among children under five (Tazinya et al., 2018), highlighting the urgent need for effective prevention and control strategies. ARTIs are caused primarily by viral and bacterial pathogens (Yang et al., 2022). Environmental and host factors further contribute to susceptibility and severity. Viral pathogens are the leading cause, with common agents including respiratory syncytial virus (RSV), parainfluenza virus (PIV), coronavirus (CoV), and adenovirus (ADV) (Clementi et al., 2021). The SARS-CoV-2 virus, as the causative agent of COVID-19, led to a global pandemic, resulting in over 774 million confirmed cases and more than 7 million deaths worldwide by January 7, 2024 (World Health Organization, 2024). Bacterial pathogens can also cause ARTIs. Common bacterial pathogens include *Chlamydia pneumoniae* (CP), *Mycoplasma pneumonia* (MP), *Legionella pneumophila* (LP), and *Coxiella burnetii* (CB) (Garin et al., 2002; Blasi, 2004).

The transmission of respiratory pathogens, primarily through droplets, is marked by its considerable infectious potential, rapid spread, and the tendency to cause localized outbreaks (Pöhlker et al., 2023). The prevalence of respiratory pathogens is influenced by a range of factors, including seasonal variation, geographic and socioeconomic conditions, population characteristics, and public health measures and hygiene practices (Chan et al., 2023; Tang et al., 2023; Dallmeyer et al., 2024). Together, these factors impact infection patterns and severity, particularly among vulnerable populations. Considering regional variability, studying pathogen epidemiology at the local level is essential for developing targeted health strategies and enabling rapid outbreak response.

Non-pharmaceutical interventions (NPIs), including social distancing, hand washing, mask-wearing, isolation, travel restrictions, and lockdowns, have been widely implemented to mitigate SARS-CoV-2 transmission during the COVID-19 pandemic. Studies show that NPIs led to a decline in pediatric ARTI cases, alleviating pressure on healthcare systems but potentially causing “immunity debt” by reducing routine viral exposure (Ladhani and Harker, 2021). Furthermore, changes in pathogen prevalence driven by NPIs may lead to the emergence of new pandemics. Researching pathogen epidemiology before and after the implementation NPIs is essential to assess their effectiveness, understand their broader impact on infectious agents, inform future health strategies, and monitor for a pandemic emergence.

Lanzhou is a large inland city on the vast Loess Plateau in northwestern China. It has a temperate continental climate characterized by arid conditions, limited precipitation, and low per capita income (Zhang et al., 2023). In Lanzhou, NPIs were implemented in January 2020, and were lifted in December 2022. In this context, our study, conducted from July 2019 to January 2024 in Lanzhou, investigates the characteristics and epidemiological trends of respiratory pathogens in children with ARTIs. The findings aim to inform the strategies for the prevention, control, and management of respiratory infections in Gansu Province. As the only comprehensive pediatric facility in the province, the Gansu Provincial Children’s Hospital, affiliated with the Lanzhou University Second Hospital, integrates healthcare services, educational programs, research initiatives, and preventive measures. With 274 beds, the hospital is a vital healthcare resource, averaging 140,000 outpatient and emergency visits, over 10,000 pediatric hospitalizations, and more than 2,000 surgical procedures annually. The data collected from this study provide a comprehensive view of the respiratory pathogen landscape in the Lanzhou area, contributing significantly to the understanding and management of ARTIs in the region.

## Materials and methods

### Study participants

Children (≤18 years) diagnosed with ARTIs between July 2019 and January 2024 were included in the study. The inclusion criteria were: (i) the presence of fever along with one or more symptoms such as cough, nasal congestion, rhinorrhea, sore throat, expectoration, or headache; and (ii) testing for respiratory pathogens. Patients with incomplete clinical records were excluded. To ensure data accuracy and prevent duplication, specific criteria were applied to manage multiple hospital visits. For each ARTI episode, only the first hospital visit was included if multiple visits occurred within a 6–8 week period with consistent symptoms and pathogen results. Repeat visits within this timeframe with no change in symptoms or pathogen results were excluded. However, if a new visit occurred more than 8 weeks after the last confirmed ARTI diagnosis, with new or worsened symptoms or a different pathogen detected, it was classified as a new infection episode. Participants were categorized based on their age into five groups: neonates (age <1 month), infants (1 month≤age<1 year), toddlers (1 year≤age<3 years), preschool children (3 years≤age<7 years), and school-aged children (7 years≤age ≤ 18 years).

### Specimen collection and respiratory pathogen detection

This study analyzed existing clinical data from indirect immunofluorescence (IIF) tests conducted as part of routine diagnostics at Lanzhou University Second Hospital. On the day of consultation or hospitalization, 2ml of peripheral venous blood was collected from the children and placed in a procoagulant tube. To prevent contamination from skin flora, aseptic operation was strictly carried out during the blood collection process. The blood specimens were kept at room temperature and centrifuged within 2 hours for 10 minutes at 3400 r/min (with a radius of 10 cm and a force of 1500×g). The upper serum layer was then extracted and stored in a refrigerator at 4-6°C, with testing completed within 24 hours. The respiratory pathogens studied included influenza A virus (FluA), influenza B virus (FluB), parainfluenza virus (PIV), respiratory syncytial virus (RSV), adenovirus (ADV), *Mycoplasma pneumonia* (MP), *Chlamydia pneumoniae* (CP), *Coxiella burnetii* (CB), and *Legionella pneumophila* (LP), detected by indirect immunofluorescence assay. Detection was performed using the Respiratory Pathogen Spectrum Antibody IgM Detection Kit (EUROIMMUN, Germany).The experimental procedure strictly followed the manufacturer’s instructions and adhered to standard laboratory operating procedures.

### Statistical analysis

Data for this study were extracted from the laboratory information system (LIS) using the Donghua software platform. Categorical variables were analyzed and presented as counts (n) and percentages (%). Pearson’s chi-square test was applied to assess the statistical significance of differences among the variables, with Fisher’s exact test used when data distribution or specific conditions required it. Additionally, analysis of variance (ANOVA) was used to compare group means. All statistical analyses were conducted in SPSS 25.0 (IBM Inc., Chicago, IL, USA),with P < 0.05 was considered statistically significant.

## Results

### Participant demographics and enrollment overview

**Figure 1:**
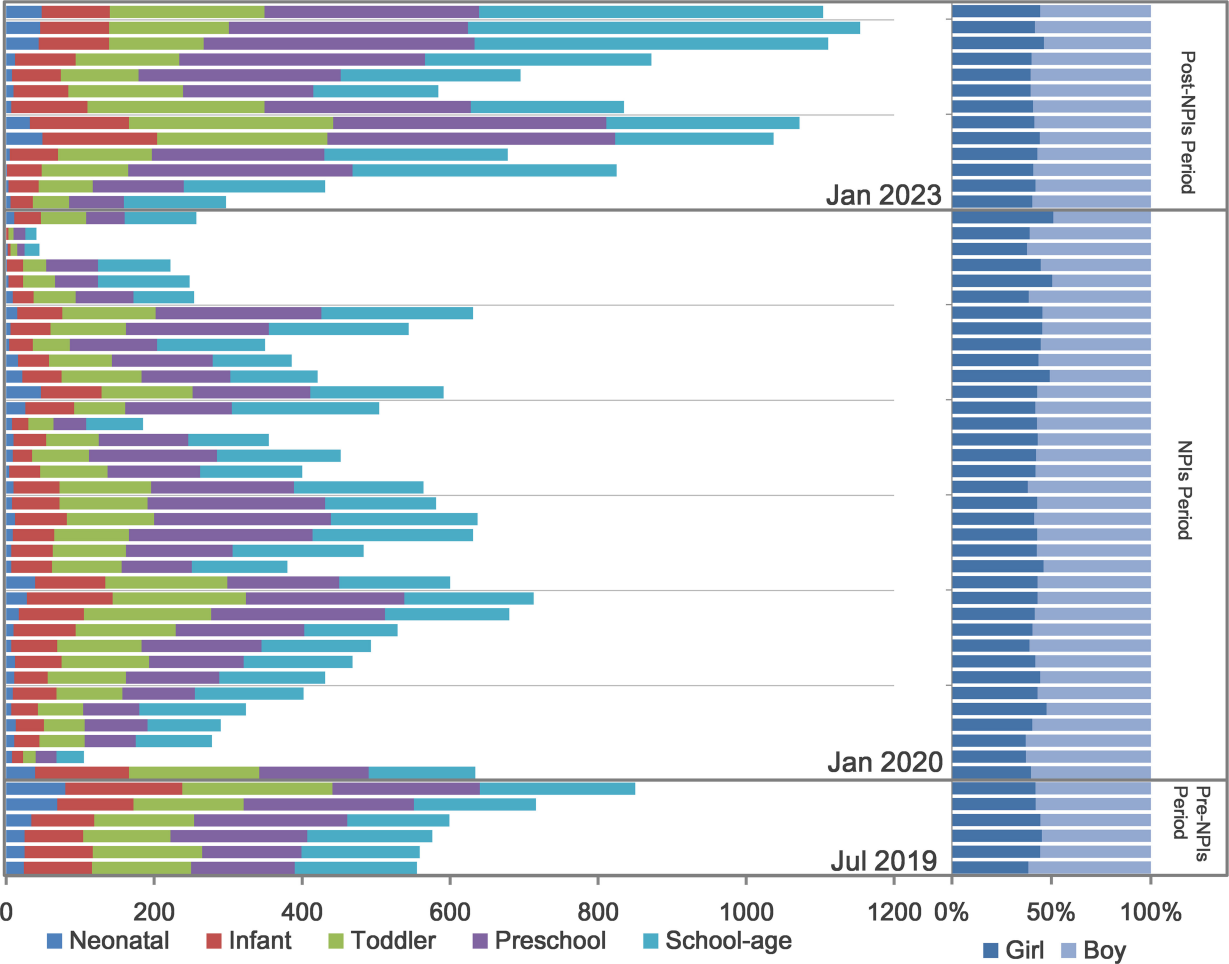
Children with ARTIs tested for respiratory pathogens from July 2019 to January 2024. The composite bar chart on the left shows the number of children tested monthly alongside the distribution across different age groups. The 100% stacked bar chart on the right shows the relative proportions of male and female children. NPIs: Non-pharmaceutical Interventions. Pre-NPIs: July 2019 to December 2019, NPIs: January 2020 to December 2022, post-NPIs: January 2023 to January 2024. Neonates: age <1 month, infants: 1 month≤age<1 year, toddlers: 1 year≤age<3 years, preschool children: 3 years≤age<7 years, school-aged children: 7 years≤age ≤ 18 years.

This study included 29,659 pediatric patients diagnosed with ARTIs. Annual enrollment varied, with 3,855 patients enrolled from July to December 2019, 5,347 in 2020, 5,772 in 2021, 3,990 in 2022, peaking at 9,591 in 2023, and 1,104 in January 2024. The gender distribution was 17,041 males and 12,618 females (male-to-female ratio 1.35:1). In terms of age demographics, the study comprised 985 neonates, 3,548 infants, 6,145 toddlers, 9,319 preschoolers, and 9,662 school-aged children, with a median age of 4.0 years(range:1 day to 18 years,mean: 4.97years, SD ± 4.20) (**Figure 1**).

### Detection rates of respiratory pathogens by pathogen type, gender, and age in pediatric ARTI cases

**Figure 2:**
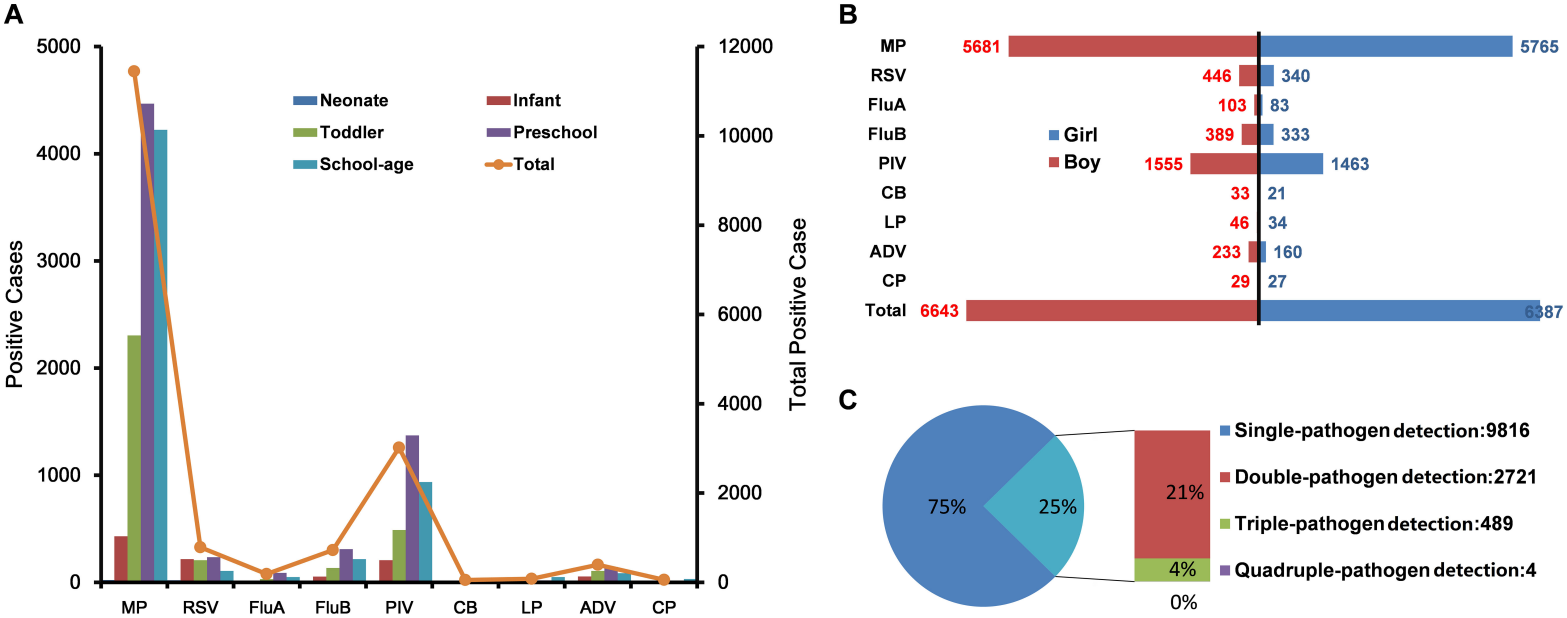
Prevalence of respiratory pathogen detection from July 2019 to January 2024. **(A)** Positive cases identified among different respiratory pathogens and across various age groups. **(B)** Positive cases of various respiratory pathogens identified in children of different sexes. **(C)** Relative proportions and case counts of single-pathogen versus multi-pathogen detection. Single-pathogen detection: Cases with only one pathogen, double-pathogen detection: cases with two pathogens, triple-pathogen detection: cases with three pathogens, quadruple-pathogen detection: Cases with four pathogens. MP, mycoplasma pneumonia; RSV, respiratory syncytial virus; FluA, influenza A virus; FluB, influenza B virus; PIV, parainfluenza virus; CB, Coxiella burnetii; LP, Legionella pneumophila; ADV, adenovirus; CP,Chlamydia pneumonia. Neonates: age <1 month, infants: 1 month≤age<1 year, toddlers: 1 year≤age<3 years, preschool children: 3 years≤age<7 years, school-aged children: 7 years≤age ≤ 18 years.

MP had the highest detection rate at 38.59%, followed by PIV at 10.18%. Detection rates for other pathogens were lower, including RSV at 2.65%, FluB at 2.43%, ADV at 1.33%, FluA at 0.63%, LP at 0.27%, CP at 0.19%, and CB at 0.18%. Out of 29,659 cases, 43.93% (13,030/29,659) tested positive for at least one respiratory pathogen, with single-pathogen detections comprising 75.33% (9,816/13,030) and multi-pathogen detections making up 24.67% (3,214/13,030). Most multi-pathogen cases involved two pathogens (84.66%, 2,721/3,214), with MP and PIV being the most common combination (52.96%,1,702/3,214), followed by MP and FluB (13.75%,442/3,214). Triple-pathogen cases were less common (15.21% 489/3,214), with MP, RSV, and PIV as the most frequent combination (47.03%,230/489), quadruple-pathogen infections were rare (0.12%,4/3,214) (**Table 1**; **Figures 2A, C**; **Supplementary Table S1**).

**Table 1:** Detection rates of respiratory pathogens by pathogen type, gender, and age distribution in pediatric ARTI cases.

	MP	RSV	FluA	FluB	PIV	CB	LP	ADV	CP
Respiratory Pathogen Detection rate: n(%)									
single-pathogen detection: 9816 (33.10)	8397 (28.31)	215 (0.72)	34 (0.11)	197 (0.66)	789 (2.66)	31 (0.10)	41 (0.14)	82 (0.28)	30 (0.10)
double-pathogen detection: 2721 (9.17)	2560 (8.63)	284 (0.96)	93 (0.31)	457 (1.54)	1827 (6.16)	21 (0.07)	36 (0.12)	145 (0.49)	19 (0.06)
triple-pathogen detection: 489 (1.65)	485 (1.64)	285 (0.96)	57 (0.19)	66 (0.22)	398 (1.34)	2 (0.01)	3 (0.01)	166 (0.56)	5 (0.02)
quadruple-pathogen detection: 4 (0.01)	4 (0.01)	2 (0.01)	2 (0.01)	2 (0.01)	4 (0.01)	0 (0.00)	0 (0.00)	0 (0.00)	2 (0.01)
Total: 13030 (43.93)	11446 (38.59)	786 (2.65)	186 (0.63)	722 (2.43)	3018 (10.18)	54 (0.18)	80 (0.27)	393 (1.33)	56 (0.19)
P-value	0.000	0.000	0.000	0.000	0.000	0.013	0.000	0.000	0.000
Gender distribution rate: n(%)									
male: 6643 (38.98)	5681 (33.34)	446 (2.62)	103 (0.60)	389 (2.28)	1555 (9.13)	33 (0.19)	46 (0.27)	233 (1.37)	29 (0.17)
female: 6387 (50.62)	5765 (45.63)	340 (2.69)	83 (0.66)	333 (2.64)	1463 (11.61)	21 (0.17)	34 (0.27)	160 (1.27)	27 (0.21)
P-value	0.000	0.682	0.565	0.049	0.000	0.587	0.994	0.460	0.390
Age distribution rate: n(%)									
Neonate: 58 (5.9)	22 (2.20)	20 (2.00)	0 (0.00)	9 (0.90)	15 (1.50)	0 (0.00)	0 (0.00)	3 (0.30)	1 (0.1.)
Infant: 767 (21.62)	430 (12.12)	217 (6.11)	17 (0.48)	53 (1.49)	207 (5.84)	4 (0.11)	1 (0.03)	55 (1.55)	0 (0.00)
Toddler: 2611 (42.47)	2304 (37.50)	207 (3.37)	30 (0.49)	134 (2.18)	488 (7.94)	10 (0.16)	12 (0.20)	106 (1.73)	8 (0.13)
Preschool: 4984 (53.45)	4467 (47.95)	235 (2.52)	89 (0.96)	310 (3.33)	1372 (14.72)	14 (0.15)	18 (0.19)	144 (1.55)	16 (0.17)
School-age: 4610 (47.70)	4223 (43.69)	107 (1.11)	50 (0.52)	216 (2.24)	936 (9.68)	26 (0.27)	49 (0.51)	85 (0.88)	31 (0.32)
P-value	0.000	0.000	0.000	0.000	0.000	0.116	0.000	0.000	0.002

n (%): the count and percentage of cases. Single-pathogen detection: Cases with only one pathogen, double-pathogen detection: cases with two pathogens, triple-pathogen detection: cases with three pathogens, quadruple-pathogen detection: Cases with four pathogens. MP, mycoplasma pneumonia; RSV, respiratory syncytial virus; FluA, influenza A virus; FluB, influenza B virus; PIV, parainfluenza virus; CB, Coxiella burnetii; LP, Legionella pneumophila; ADV, adenovirus; CP,Chlamydia pneumonia.

Gender differences in pathogen detection were observed, with higher cumulative detection rates in female children than male children (50.62% vs. 38.98%, P < 0.001). MP and PIV were detected more frequently in females than males (MP: 45.63% vs. 33.34%; PIV: 11.61% vs. 9.13%; P < 0.001). FluB also showed a slightly higher prevalence in females than males (2.64% vs. 2.28%, P < 0.05) (**Figure 2B**).

Significant age-based differences in pathogen detection rates were observed (P < 0.001). Pathogen prevalence was generally higher in preschool-aged children, with MP reaching a peak detection rate of 47.95% and PIV at 14.72%, respectively (P < 0.001). In contrast, neonates showed the lowest detection rates across most pathogens, with MP detected at only 5.9% and no detections of FluA or LP (P < 0.001). RSV was most frequently detected in infants (12.12%), while ADV was highest among toddlers (1.73%) (P < 0.001) (**Figure 2A**).

### Seasonal and annual variability in respiratory pathogen detection rates

**Figure 3:**
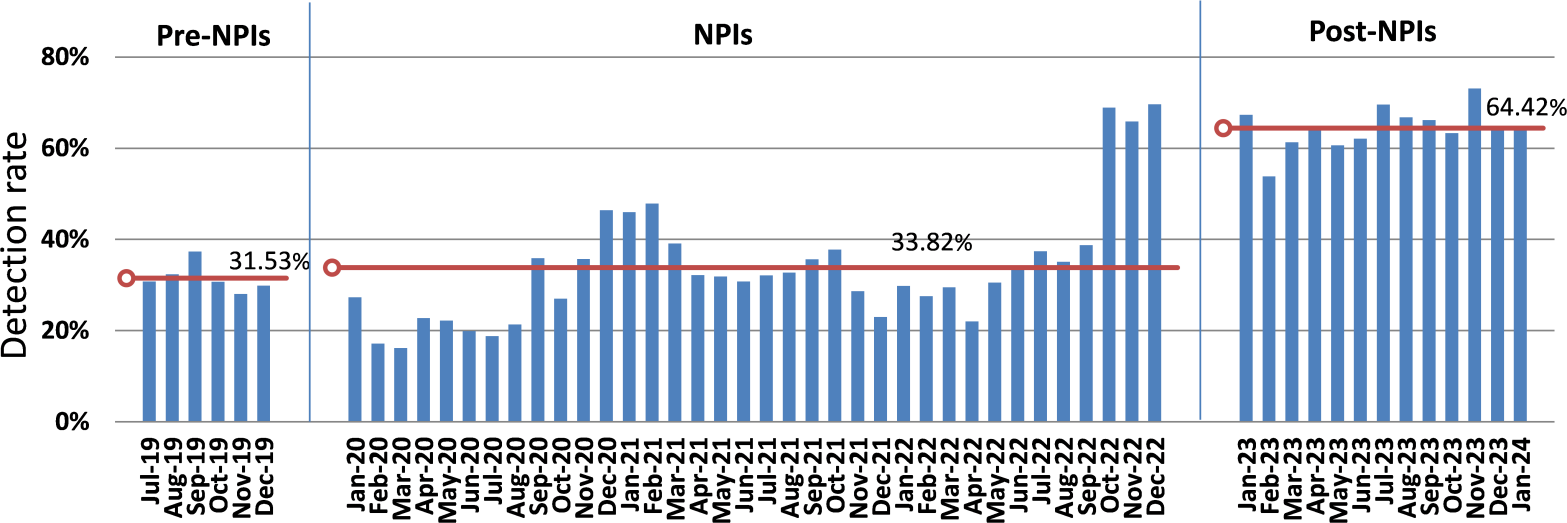
Trends in Detection Rate Across Different Intervention Phases: A Comparison of Pre-NPI, NPI, and Post-NPI Periods. The mean detection rate for each period is marked by a red line. Pre-NPIs: July 2019 to December 2019, NPIs: January 2020 to December 2022, post-NPIs: January 2023 to January 2024.

Specifically, detection rates in the colder and drier second half of the year (July to December) increased consistently across the years, from 31.28% in 2019 to 67.42% in 2023 (P < 0.05). When analyzing full-year detection rates, a progressive increase was observed from 2020 to 2023: 28.60% in 2020, 34.79% in 2021, 34.31% in 2022, and a peak of 64.76% in 2023 (P < 0.001). Based on the climate classification in Lanzhou, February to April corresponds to spring, May to July to summer, August to October to autumn, and November to January to winter. Winter consistently showed the highest detection rate among all seasons (47.61%), followed by summer (43.05%), autumn (42.72%), and spring (41.57%) (P < 0.001). The winter of 2022 registered the highest seasonal detection rate at 68.24%, which was significantly elevated compared to other seasons (P < 0.001), while the spring of 2020 displayed the lowest rate at 19.17% (P < 0.001). Monthly analysis further revealed that November had the highest average detection rate (48.92%), while August recorded the lowest (39.38%) (P < 0.001) (**Figure 3**).

### Comparison of detection rates before, during, and after NPIs

**Figure 4:**
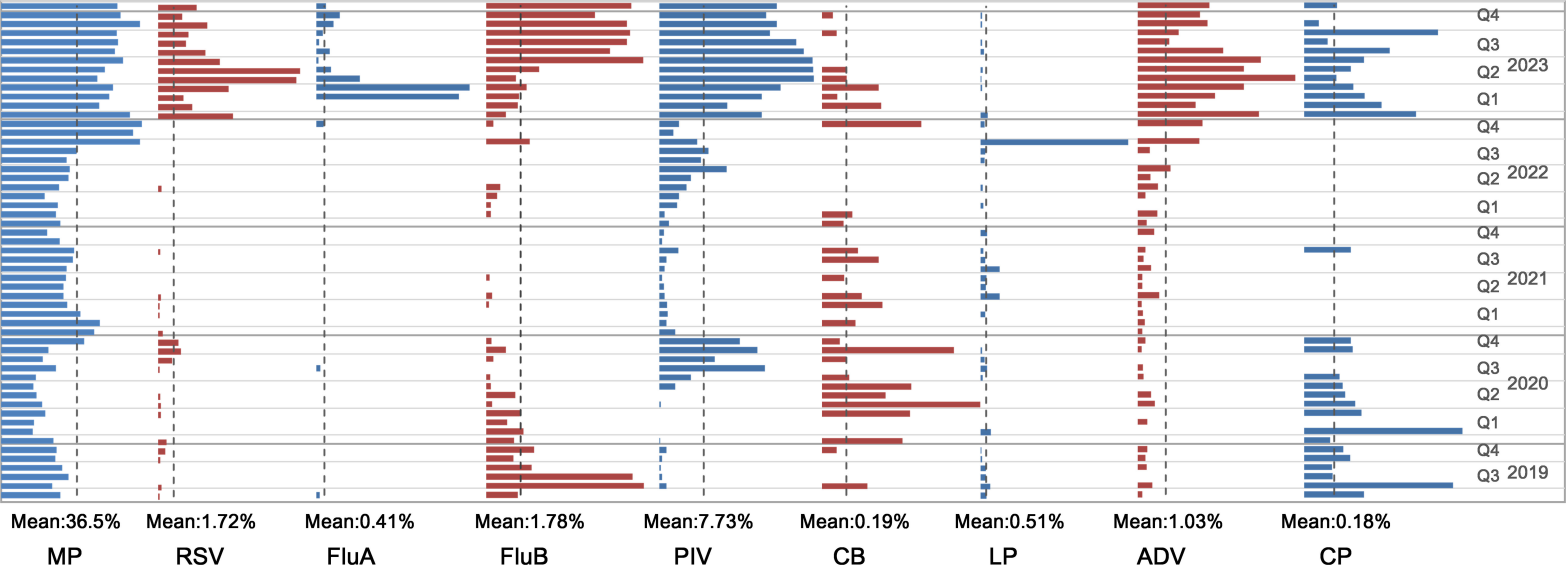
Monthly prevalence of individual pathogen in percentage of specimens. The mean value of each pathogen is marked by dotted line. MP, mycoplasma pneumonia; RSV, respiratory syncytial virus; FluA, influenza A virus; FluB, influenza B virus; PIV, parainfluenza virus; CB, Coxiella burnetii; LP, Legionella pneumophila; ADV, adenovirus; CP,Chlamydia pneumonia.

There are significant differences in mean monthly detection rates across the three periods (P < 0.001).During the pre-NPIs Period, data was available only from July to December 2019. Therefore, the analysis for this period is based on these six months, with a mean monthly detection rate of 31.53% (SD ± 3.17). The subsequent NPIs Period showed a slightly higher mean detection rate at 33.82% (SD ± 13.13). However, it was in the Post-NPIs Period, after the lifting of these interventions, a marked increase in detection rates was observed, rising to 64.42% (SD ± 4.67).November 2023 recorded the highest detection rate (73.09%). Significant fluctuations were observed in both the testing cases and the detection rates in certain months. In February 2020, the testing cases decreased dramatically from 634 in the preceding month to 105, with the detection rate also falling from 27.29% to 17.14%. Subsequently, starting in September of the same year, the number of testing cases recovered, ranging from 355 to 713, with detection rates varying between 27.03% and 46.42%. From November 2021 to April 2022, the detection rate remained comparatively low, staying below 30%. In July 2022, the testing cases decreased from 631 in the previous month to 254, and continued to decline thereafter. By November 2022, only 41testing cases, but the detection rate surged to 65.85%. Except for February 2023, which had a detection rate of 53.83%, the subsequent months consistently showed detection rates exceeding 60%, with the number of tested individuals ranging from 257 to 1154 (**Figures 1**, **3**, **4**).

The implementation of NPIs had a significant suppressive effect on respiratory pathogen transmission, with a notable rebound effect observed after their removal. MP was the most frequently detected pathogen each month, with its detection rate sharply increasing from 36.49% in the previous month to 66.67% in October 2022, and remaining above 45% thereafter. PIV maintained prevalence throughout the year, spiking from 3.50% in December 2022 to 17.85% in January 2023, and stabilizing around 20% after April. RSV and ADV showed low detection rates, occasionally reaching 0% before December 2022. However, RSV’s detection rate surged to 8.08% in January 2023, then increased to 14.95% in May and 15.39% and June. ADV peaked at 4.38% in January 2023. FluA was virtually undetected from July 2019 until it spiked to 7.27% in March and 7.82% in April 2023. FluB showed higher detection rates in August(8.05%) and September 2019(7.47%), which decreased after October(2.34%), before increased again in July 2023(8.02%).CB and LP had consistently low detection rates, but LP surged to 13.33% in October 2022. CP generally had very low monthly detection rates, which increased from 0% in prior months to 0.67% in January 2023 (**Figure 4**).

## Discussion

Our analysis investigated the prevalence of respiratory pathogens in 29,659 pediatric patients with ARTIs in Lanzhou, Northwest China, from July 2019 to January 2024. This study coincided with the COVID-19 pandemic and the implementation of NPIs in Lanzhou to curb the spread of SARS-COV-2, providing a unique context to evaluate pathogen prevalence rates. By navigating the complexities of respiratory pathogen infections amid these global health challenges, our analysis seeks to offer invaluable insights into the dynamics of pediatric ARTIs, contributing to the ongoing discourse on effective public health strategies and interventions.

Our analysis reveals that the pathogen detection rate in the Lanzhou region not only surpasses those reported in Suzhou(2006-2015, 30.74%) (Yinying et al., 2019), Beijing(2020,41.71%) (Gu et al., 2022), and Shanghai(2015-2019,36.96%) (Xu et al., 2022), but also shows a significant increase from a previous study in Lanzhou (2015,37.80%) (Zhang et al., 2016). However, it was lower than the rates reported in Ningbo (2019-2021, 56.17%) (Zhou et al., 2022) and in Suzhou (2005-2021, 57.32%) (Ji et al., 2013). This suggests that the prevalence of respiratory pathogens is influenced by factors such as the surveillance year, geographic location, local climatic conditions, community living habits, and methodological approaches to pathogen detection. Additionally, the variance across regions highlights the need for region-specific public health strategies that account for local environmental and social factors.

In this study, the detection rate in female children (49.92%) was higher than in male children (38.16%), consistent with previous reports from Lanzhou (2021-2022, 19.31% vs. 15.65%) (Wang et al., 2023) and data from Beijing (2020, 47.26% vs. 37.56%) (Gu et al., 2022). Conversely, studies from Ningbo (Zhou et al., 2022) and Guangzhou (Xie et al., 2023) (2019-2021, 56.92% vs. 43.08%; 2018-2021, 47.40% vs. 44.95%) reported higher detection rates in male children. Notably, a study from Huzhou (Juan et al., 2015) (2011-2013) found no gender difference in respiratory pathogen detection. Additionally, the number of boys who underwent respiratory pathogen testing was significantly higher than that of girls (17,041 vs. 12,618). The reasons for these differences could relate to the year of monitoring, geographic area, local population’s awareness, or the local child population’s gender ratio. In Lanzhou, 52.39% of the child populations are boys, while 47.61% are girls. Some studies have suggested that access to medical resources or health-seeking behavior may vary by gender, potentially contributing to the observed differences in detection rates (Zhang et al., 2016; Al Kindi et al., 2023).

Our findings revealed age-related differences in pathogen detection rates among pediatric patients with ARTIs, with newborns and infants exhibiting lower rates compared to preschool and school-aged children. This pattern likely reflects a combination of immunological, social, and behavioral factors (Prescott et al., 2021; Zhang et al., 2021; Sorci and Faivre, 2022; Tang et al., 2023). Newborns and infants benefit from maternal antibodies transferred via the placenta and breast milk, providing passive immunity that offers initial protection against many infections (Apostol et al., 2020; Langel et al., 2020; Rad et al., 2021). During the COVID-19 pandemic, research showed that lactating mothers vaccinated against SARS-CoV-2 produced antibodies (IgG1, IgA, IgM) present in breast milk, potentially providing protection against the virus to their breastfeeding infants (Quitadamo et al., 2021; Demers-Mathieu, 2023). Additionally, their limited social interactions reduce their exposure to infectious agents. In contrast, preschool and school-aged children, engaging more frequently in group activities and attending educational settings, face increased exposure to pathogens. The expanding social networks and engagement with the external environment in this age group heighten their risk of exposure to infectious agents. As immune system matures with age, shifting from passive immunity to more active responses (Firth et al., 2005), this progression may also influence detection rates. To mitigate the higher infection risk faced by preschool and school-aged children, public health interventions could focus on enhancing hygiene practices within educational settings and adjusting vaccination schedules to provide better protection against prevalent pathogens.

The observed variations in the distribution of respiratory pathogens among children in the Lanzhou region from 2019 to 2024 highlight the combined effects of natural seasonal fluctuations and the significant impact of public health interventions implemented in response to the COVID-19 pandemic. NPIs were began to implement in Lanzhou in early 2020 due to the COVID-19 pandemic. These interventions included measures such as mask-wearing, pausing offline education, and reducing people’s movement and gatherings. On February 21, 2020, Gansu’s COVID-19 emergency response level was lowered from level one to level three. Schools reopened, movement and gatherings became more frequent, and protective measures were relaxed, leading to a decrease in the public’s protection awareness. Lanzhou experienced several significant COVID-19 outbreaks in October and November 2021, March and April 2022, July and August 2022, and again in October and November 2022. During these periods, the government strengthened control measures to combat the spread of the virus. The implementation of these epidemic prevention and control measures reduced transmission opportunities for respiratory pathogens on one hand (Taktak et al., 2023), and on the other hand, decreased the number of individuals seeking medical attention for respiratory infections (Haddadin et al., 2021; Mataloni et al., 2022). In December 2022, the lifting of most pandemic restrictions in Lanzhou led to a noticeable increase in the detection rate. This phenomenon is consistent with a global trend: as restrictions ease, the detection and spread of respiratory pathogens tend to increase (Al Kindi et al., 2023; Chan et al., 2023). The fluctuations observed in the monthly testing volumes and detection rates of respiratory pathogens are closely aligned with the adjustments in NPIs measures. During periods of intensified control measures—such as school closures—there was a notable reduction in both testing numbers and detection rates. This decrease can be partly attributed to the reduced interactions among children, which in turn lowered the risk of transmitting ARTIs. Additionally, during the COVID-19 pandemic, parental concerns about hospital-based infections led to hesitancy in seeking face-to-face medical care for their children (Radhakrishnan, 2022), with many choosing to delay treatment for respiratory symptoms or resorting to online medical consultations. This shift not only reduced the number of tests conducted but also likely influenced trends in the spread and detection of respiratory pathogens.

The high detection rate of MP in our study aligns with findings from global studies, which also identify MP as a highly prevalent pathogen (Kumar, 2018; Kutty et al., 2019; Lee et al., 2020). PIV had the second highest detection rate. In this study, Co-detection of MP and PIV (52.96%) was the most common among multi-pathogen cases, Reports also indicate that MP is the most frequently detected pathogen in co-infections with PIV type 3 (PIV3) (Lu et al., 2023).This study further underscores the variability in the distribution of pathogens in pediatric respiratory infections, highlighting differences across geographic regions. For instance, in Ningbo, RV had the highest detection rate(24.05%), followed by RSV(10.45%) and MP(7.03%) (Zhou et al., 2022). In contrast, the Guangzhou reported RSV(13.75%), ADV(4.82%), and PIV(4.82%) as the most prevalent pathogens (Xie et al., 2023).The onset of pandemic and subsequent NPIs have distinctly impacted the seasonal epidemiology of respiratory pathogens transmitted via droplet or contact transmission, such as MP,PIV, RSV, ADV, FluA, FluB, and CP. After January 2020, the usual seasonal trends of these pathogens were markedly disrupted, with many showing atypical patterns or a significant decline in detection rates. With the lifting of NPIs in December 2022, a marked resurgence in detection rates was observed, highlighting their considerable impact on pathogen transmission dynamics. Conversely, pathogens such as CB and LP, which rely on alternative transmission modes—LP via water systems (World Health Organization; Barskey et al., 2022; Dowdell et al., 2023) and CB through inhalation of dust contaminated with infected animal birthing products, feces, urine, or milk (Anderson et al., 2013)—did not show significant epidemiological shifts during this period. This stability suggests that pathogens with unique environmental vectors may have been less directly affected by the social and behavioral changes induced by NPIs. The observed surge in the detection rate of respiratory pathogens, as well as the overall increase in ARTIs among children after December 2022, can largely be attributed to the prolonged implementation of NPIs during the COVID-19 pandemic. While these measures were crucial for controlling the spread of COVID-19, they disrupted the typical seasonal patterns of pathogen prevalence at the same time. As social distancing, mask-wearing, and lockdowns reduced exposure to common pathogens, a significant portion of the population, particularly children, may have missed opportunities for natural immunization. Consequently, these interventions resulted in the accumulation of a susceptible population, leading to an uptick in ARTI cases in 2023.This situation underscores the delicate balance between immediate public health needs and long-term epidemiological trends. While NPIs were essential in managing COVID-19 transmission, their broader impacts on the epidemiology of other respiratory pathogens highlight the importance of integrating pandemic response strategies with efforts to maintain population immunity against diverse infectious agents.

## Limitations

It should be noted that this study can only identify associations between variables, but not establish causality. Given that this research focuses exclusively on children with ARTIs and employs the IIF test, the findings may not be generalizable to other demographic groups or samples analyzed using alternative testing methods. Additionally, this study may not accurately capture all clinical characteristics. The study use clinical IIF test data, constrained by the limitations of the testing kits, do not encompass all pathogens. Factors like disease progression, nutrition, and immune status can influence antibody production, potentially leading to an underestimation of some pathogens’ prevalence. The data collected span periods during and after NPIs of the COVID-19 pandemic, and consequently, the study’s findings are likely to reflect the regional epidemic trends specific to this period. Lastly, as the data originates from only one hospital, more extensive data collection across Lanzhou is needed to accurately characterize the epidemiology of respiratory pathogens.

## Conclusion

In conclusion, this study has characterized the epidemiological landscape of respiratory pathogens among pediatric patients with ARTIs in Lanzhou, China, from 2019 to 2024, a period marked by significant public health challenges due to the COVID-19 pandemic. Respiratory pathogens exhibited a seasonal peak in the winter months, with MP and PIV identified as the most prevalent among children with ARTIs, females and preschool-aged children showed higher susceptibility. NPIs significantly reduced respiratory pathogen transmission; however, their removal led to a notable resurgence, particularly in MP and PIV cases. These findings not only enhance our understanding of respiratory infection dynamics during a critical period but also highlight the need for public health strategies and clinical practices specifically tailored to the pediatric population. As we move forward, it is imperative to continue monitoring these epidemiological trends, using the insights gained to strengthen preventive and therapeutic measures against respiratory infections. Future research should aim to address the limitations of this study and explore the evolving landscape of respiratory pathogens in the wake of the COVID-19 pandemic and beyond.

## Data Availability

The original contributions presented in the study are included in the article/**Supplementary Material**. Further inquiries can be directed to the corresponding authors.

## Supplementary material

Supplementary Table 1 Cases of multi-pathogen detection from July 2019 to January 2024, detailing the specific cases of double-pathogen, triple-pathogen, and quadruple-pathogen infections involving different respiratory pathogens.
