# Supplementary material for: Analysis of respiratory pathogens in pediatric acute respiratory infections in Lanzhou, Northwest China, 2019-2024
Source: Front Cell Infect Microbiol. 2025 Jan 17;14:1494166. doi: 10.3389/fcimb.2024.1494166 (PMC11782233; doi:10.3389/fcimb.2024.1494166)
Supplement: Supplementary Table 1 — Cases of multi-pathogen detection from July 2019 to January 2024, detailing the specific cases of double-pathogen, triple-pathogen, and quadruple-pathogen infections involving different respiratory pathogens. [file Table1.docx]

Table S1. Multi -pathogen detection cases from July 2019 to January 2024.

|  | Double-pathogen Detection | | | | | | | |  | Triple-pathogen Detection | | | | | | |  | Quadruple-pathogen Detection | | |
| --- | --- | --- | --- | --- | --- | --- | --- | --- | --- | --- | --- | --- | --- | --- | --- | --- | --- | --- | --- | --- |
|  | RSV | FluA | FluB | PIV | CB | LP | ADV | CP |  | RSV&FluA,FluB,PIV,CB,LP,ADV,CP | FluA&FluB,PIV,CB,LP,ADV,CP | FluB&PIV,CB,LP,ADV,CP | PIV&CB,LP,ADV,CP | CB&LP,ADV,CP | LP&ADV,CP | ADV&CP |  | FluA&FluB&PIV | RSV&FluB&PIV | FluA&PIV&CP |
| MP | 182 | 70 | 442 | 1702 | 18 | 33 | 98 | 15 |  | 8,28,230,0,0,16,1 | 3,38,0,1,4,0 | 6,0,0,29,0 | 2,2,113,3 | 0,0,0 | 0,0 | 1 |  | 1 | 1 | 1 |
| RSV |  | 10 | 7 | 74 | 1 | 0 | 10 | 0 |  |  | 0,1,0,0,0,0, | 0,0,0,0,0 | 0,0,1,0 | 0,0,0 | 0,0 | 0 |  |  |  |  |
| FluA |  |  | 1 | 11 | 0 | 0 | 1 | 0 |  |  |  | 0,0,0,0,0 | 0,0,2,0 | 0,0,0 | 0,0 | 0 |  |  |  |  |
| FluB |  |  |  | 1 | 0 | 0 | 3 | 3 |  |  |  |  | 0,0,0,0 | 0,0,0 | 0,0 | 0 |  |  |  |  |
| PIV |  |  |  |  | 2 | 3 | 33 | 1 |  |  |  |  |  | 0,0,0 | 0,0 | 0 |  |  |  |  |
| CB |  |  |  |  |  | 0 | 0 | 0 |  |  |  |  |  |  | 0,0 | 0 |  |  |  |  |
| LP |  |  |  |  |  |  | 0 | 0 |  |  |  |  |  |  |  | 0 |  |  |  |  |
| ADV |  |  |  |  |  |  |  | 0 |  |  |  |  |  |  |  |  |  |  |  |  |
